# Psychological processes in young bullies versus bully‐victims

**DOI:** 10.1002/ab.21701

**Published:** 2017-02-08

**Authors:** Anouk van Dijk, Astrid M. G. Poorthuis, Tina Malti

**Affiliations:** ^1^ Department of Psychology Utrecht University Utrecht The Netherlands; ^2^ Research Institute of Child Development and Education University of Amsterdam Amsterdam The Netherlands; ^3^ Department of Psychology University of Toronto Mississauga Ontario Canada

**Keywords:** bullying, early childhood, emotions, social cognition, victimization

## Abstract

Some children who bully others are also victimized themselves (“bully‐victims”) whereas others are not victimized themselves (“bullies”). These subgroups have been shown to differ in their social functioning as early as in kindergarten. What is less clear are the motives that underlie the bullying behavior of young bullies and bully‐victims. The present study examined whether bullies have proactive motives for aggression and anticipate to feel happy after victimizing others, whereas bully‐victims have reactive motives for aggression, poor theory of mind skills, and attribute hostile intent to others. This “distinct processes hypothesis” was contrasted with the “shared processes hypothesis,” predicting that bullies and bully‐victims do not differ on these psychological processes. Children (*n* = 283, age 4–9) were classified as bully, bully‐victim, or noninvolved using peer‐nominations. Theory of mind, hostile intent attributions, and happy victimizer emotions were assessed using standard vignettes and false‐belief tasks; reactive and proactive motives were assessed using teacher‐reports. We tested our hypotheses using Bayesian model selection, enabling us to directly compare the distinct processes model (predicting that bullies and bully‐victims deviate from noninvolved children on different psychological processes) against the shared processes model (predicting that bullies and bully‐victims deviate from noninvolved children on all psychological processes alike). Overall, the shared processes model received more support than the distinct processes model. These results suggest that in early childhood, bullies and bully‐victims have shared, rather than distinct psychological processes underlying their bullying behavior.

## 1 INTRODUCTION

Bullying among children occurs as early as in kindergarten and potentially has severe negative consequences (Vlachou, Andreou, Botsoglou, & Didaskalou, 2011). Young children who bully others are at risk of behavior problems, peer problems, and health problems (Wolke, Woods, Bloomfield, & Karstadt, 2000, 2001). In later childhood, these children also are at risk of poor psychosocial adjustment, including low academic achievement, lack of friendships, and psychiatric symptoms (Kumpulainen et al., 1998; Nansel et al., 2001). Given the aversive outcomes associated with bullying, it is important to better understand underlying psychological processes of bullying at an early age, as to prevent escalation in later childhood.

About 10–14% of kindergartners bully others and are also victimized themselves (labeled “bully‐victims”), whereas about 4–17% of kindergartners bully others but are not victimized themselves (labeled “bullies”; Jansen et al., 2012; Wolke, Woods, Stanford, & Schulz, 2001). Young bullies and bully‐victims have been shown to differ in their social functioning. For example, research has shown that young bully‐victims have fewer friends than their noninvolved peers, are less likely to affiliate with noninvolved classmates, and are more likely to be rejected by their classmates. In contrast, young bullies have as many friends as their noninvolved peers are equally likely to affiliate with noninvolved classmates, and seem to have a controversial status in their class: they are more likely to be rejected, but are also more likely to be popular (Farmer et al., 2010; Perren & Alsaker, 2006). Thus, bullies appear to be well‐integrated in their class at an early age, whereas bully‐victims are typically marginalized (Griffin & Gross, 2004; Vlachou et al., 2011).

### 1.1 Distinct psychological processes in young bullies versus bully‐victims

The observation that young bullies and bully‐victims have different social positions in their class has led researchers to theorize that bullying behavior in these two subgroups may be driven by distinct motives. It has been proposed that bullies are more motivated by proactive reasons, such as gaining social status or getting their way, whereas bully‐victims are more motivated by reactive reasons, such as feeling angry or repelling perceived social threats (e.g., Griffin & Gross, 2004; Olweus, 1978; Rodkin, Espelage, & Hanish, 2015; Vlachou et al., 2011). In line with this argument, researchers have suggested that certain psychological processes may underlie the bullying behavior of bullies but not bully‐victims, or vice versa (e.g., Camodeca, Goossens, Schuengel, & Terwogt, 2003; Gasser & Keller, 2009). We here investigate this “distinct processes hypothesis,” focusing on three psychological processes that are related to bullying behavior in early childhood.

First, bully‐victims (but not bullies) may have poor theory of mind skills, or a limited ability to take another person's perspective. This notion is based on conflicting findings regarding theory of mind skills in aggressive children. On the one hand, research has shown that hard‐to‐manage preschoolers with poor theory of mind skills tend to behave more negatively towards their peers (i.e., they showed more insulting, whining, and controlling behavior while playing a game; Hughes, Cutting, & Dunn, 2001). This finding suggests that children may bully others because they insufficiently comprehend their peers’ mental states. On the other hand, research has shown that a subgroup of “ringleader” bullies have intact theory of mind skills and in fact may use these skills to manipulate others (Sutton, Smith, & Swettenham, 1999). Given the distinction in social functioning between bullies and bully‐victims, it has been proposed that these conflicting findings apply to different subgroups of bullies: Poor theory of mind skills may characterize bully‐victims, but not the subgroup of socially well‐integrated bullies (Gasser & Keller, 2009).

Second, bully‐victims (but not bullies) may tend to interpret their peers’ intentions as hostile. Such “hostile intent attributions” are uniquely related to reactive aggression (Arsenio, Adams, & Gold, 2009; Dodge & Coie, 1987) and as such may characterize bully‐victims, who engage in bullying as they respond to the perceived hostility of their peers’ behavior (Camodeca et al., 2003). In contrast, bullies who are not victimized themselves may be less inclined to perceive hostility in their peers’ behavior.

Third, bullies (but not bully‐victims) may tend to anticipate feeling positive emotions after victimizing others. Such “happy victimizer emotions” are uniquely related to proactive aggression (Arsenio et al., 2009; Dodge, Lochman, Harnish, Bates, & Pettit, 1997) and as such may characterize bullies, who tend to initiate aggressive behavior as they focus on their own gains rather than the victim's feelings (Gasser & Keller, 2009). In contrast, bully‐victims may not be characterized by happy victimizer emotions, as they are victimized themselves and thus may be more likely to empathize with the victim (Menesini et al., 2003).

In sum, the distinct processes hypothesis predicts that bullies and bully‐victims may have distinct psychological processes underlying their bullying behavior, where as bullies may have proactive motives for aggression and attribute happiness to themselves as victimizer, bully‐victims may have reactive motives for aggression, poor theory of mind skills, and a tendency to attribute hostile intent (Table 1, left).

**Table 1:** Two hypotheses on psychological processes in children involved in bullying (B) versus Bullying and Victimization (BV) as compared to noninvolved children (NC)

	H_1_: Distinct processes	H_2_: Shared processes
Theory of mind errors	BV > NC, B	B, BV > NC
Hostile intent attributions	BV > NC, B	B, BV > NC
Happy victimizer emotions	B > NC, BV	B, BV > NC
Reactive motives	BV > NC, B	B, BV > NC
Proactive motives	B > NC, BV	B, BV > NC

### 1.2 Empirical evidence for distinct psychological processes in bullies versus bully‐victims

Empirical research comparing psychological processes between bullies and bully‐victims is scarce, particularly in early childhood. Hence, we here discuss research from early childhood to adolescence. To this date, the evidence for the distinct processes hypothesis is mixed, with some studies finding differences between bullies and bully‐victims but others not. For theory of mind skills, the evidence is inconsistent: One study found that bully‐victims (age 7–8) had poorer theory of mind skills than bullies (Gasser & Keller, 2009) whereas another, longitudinal study found no differences in theory of mind skills at age 5 between children who were classified as either bully or bully‐victim at age 12 (Shakoor et al., 2012). For hostile intent attributions, the evidence is inconsistent as well: One study found that bully‐victims attributed more blame to hypothetical peers than bullies (Camodeca et al., 2003), whereas another study found that bullies and bully‐victims (age 13–14) both expected hostile behavior from hypothetical peers (Ziv, Leibovich, & Shechtman, 2013). For happy victimizer emotions, the evidence for the distinct processes hypothesis is very limited: The two studies that addressed this question did not find that bullies had more happy victimizer emotions than bully‐victims, at age 7–8 (Gasser & Keller, 2009) nor at age 12–18 (Perren, Gutzwiller‐Helfenfinger, Malti, & Hymel, 2012). Last, the evidence for distinct aggression motives in bullies and bully‐victims as well is very limited: Four studies found that bullies and bully‐victims both had reactive *and* proactive motives for aggression, at age 7–8 (Camodeca, Goossens, Terwogt, & Schuengel, 2002) as well as in adolescence (Bettencourt & Farrell, 2013; Ragatz, Anderson, Fremouw, & Schwartz, 2011; Salmivalli & Nieminen, 2002).

Importantly, all of the studies discussed (except Perren et al., 2012) did find distinctions in the discussed psychological processes between children involved in bullying (bullies *and* bully‐victims) and noninvolved children. Thus, these psychological processes do seem important in predicting whether children bully; however, it is unclear whether these processes distinguish bullies from bully‐victims.

### 1.3 Psychological processes in bullies versus bully‐victims: Distinct or shared?

One explanation for the limited empirical support may be that bullies and bully‐victims in fact do not have different, but rather have *shared* psychological processes underlying their bullying behavior (Table 1, right). At first sight, this “shared processes hypothesis” may seem at odds with bullies’ and bully‐victims’ different social positions in their class: Why would the subgroup of well‐integrated bullies have similar psychological processes underlying their bullying behavior as the subgroup of marginalized bully‐victims? Note, however, that bullies and bully‐victims have one important similarity: They both engage in aggressive behavior towards their peers. This behavior markedly differentiates them from children noninvolved in bullying, who may rather use social strategies such as problem‐solving or avoidance to cope with peer problems. Thus, psychological processes may not so much predict from which social position children engage in bullying (i.e., bully or bully‐victim), but rather whether children engage in bullying.

An alternative explanation for the limited empirical support may be that previous research had limitations that hindered the detection of differences between bullies and bully‐victims. Hence, the present study addresses two key limitations of previous research.

First, the studies on reactive and proactive motives used questionnaires that may have been unsuited to detect distinct patterns of reactive and proactive motives in bullies versus bully‐victims. These questionnaires consisted of items that were fixed combinations of one motive with one form of aggressive behavior (e.g., “hurts others to dominate”). As a result, children's motives could have been confounded with their actual aggressive behavior: respondents may have given high ratings to children who frequently hurt others, even though these children did not have the motive to dominate. Illustrative of this confound, such questionnaires typically yield high relations between reactive and proactive motives (*r = *.70 as found in a meta‐analysis; Polman, de Castro, Koops, van Boxtel, & Merk, 2007). To address this issue, the present study used a questionnaire that assesses children's motives independently of their actual aggressive behavior (Polman, de Castro, Thomaes, & van Aken, 2009).

Second, previous research used various methodologies to assess psychological processes and included various age groups, which makes it difficult to interpret the inconsistent findings. To address this issue, the present study tested several psychological processes within one sample, using standard paradigms to assess each process. By using such an integrative approach (Malti, 2016) we aimed to optimize the sensitivity of our research design to detect distinct psychological processes underlying the behavior of bullies versus bully‐victims.

### 1.4 The present study

In sum, previous work has supported two alternative hypotheses (Table 1). Theoretical work suggests that young bullies and bully‐victims have distinct psychological processes underlying their bullying behavior (H_1_; e.g., Griffin & Gross, 2004; Olweus, 1978; Rodkin et al., 2015; Vlachou et al., 2011). However, empirical work to this date provides limited support for this hypothesis, which may imply that young bullies and bully‐victims in fact have shared psychological processes underlying their behavior (H_2_). The present study aimed to test these contrasting hypotheses, assessing several psychological processes within one sample.

Children (age 4–9) were classified as bully, bully‐victim, or noninvolved using peer‐nominations. Children's theory of mind skills, hostile intent attributions, and happy victimizer emotions were assessed using standard vignettes and false‐belief tasks; and their reactive and proactive motives for aggression using teacher‐reports. Our hypotheses were tested using Bayesian model selection; an upcoming statistical approach that in recent years has increasingly been used by researchers in child psychology (Van de Schoot et al., 2014). The advantage of this approach is that it quantifies the amount of support from the data for each hypothesis as a coherent model (instead of testing group differences for each variable separately, as would be the case with multivariate analyses). Thus, Bayesian model selection enabled us to conduct a single test that indicates which of the two contrasting hypotheses receives most support from the data.

## 2 METHODS^1^

### 2.1 Participants

Participants were 283 children aged 4–9, recruited from five primary schools in the Netherlands (59% boys; *M*_age_ = 6.70, *SD* = 1.36; 92% European nationalities, 8% other nationalities, such as Moroccan, Surinam, and Turkish). All children received active written parental consent to participate in the study (61% consent, 32% no response, 7% no consent).

### 2.2 Procedure

Children were individually interviewed in a quiet room in their school. The session lasted 35–45 minutes and was conducted by the first author or one of eight trained research assistants (i.e., female undergraduate psychology students). Children completed three tasks in the following order: (1) hostile intent attributions; (2) theory of mind; and (3) happy victimizer emotions. Last, they completed a peer‐nomination interview to assess bullying and victimization. Teachers reported on children's reactive and proactive motives for aggression. Children received stickers to thank them for their participation; teachers received a gift card.

### 2.3 Measures

#### 2.3.1 Bully groups

Bullying and victimization were assessed with a peer‐nomination interview developed for use with kindergartners (Perren & Alsaker, 2006). Starting the interview, experimenters explained the meaning of the term bullying to children, using four drawings of different types of bullying (i.e., physical, verbal, object‐related, and exclusion; Alsaker, Nägele, Valkanover, & Hauser, 2008) and emphasizing that *repeated* portrayal of these forms of behavior is called bullying. Next, children saw a grid with photographs of their classmates and were asked to identify them all. Using this grid with photographs, children nominated (1) classmates who bully others; and the (2) victims of these bullies.

Scores for bullying and victimization were calculated as the proportion of total possible nominations in the class. This total of nominations was lower than the total number of classmates, because not all children received consent to participate in the study and because we excluded nominations from children (*n* = 10) who had poor comprehension of the interview according to experimenter‐ratings (scored on a 5‐point Likert scale after completion of the interview). We also excluded nomination data from five classes (*n = *46) that had participation rates lower than 50%. Bullying scores were significantly correlated with victimization scores (*r =*.18, *P =*.005), and teacher‐rated aggression (*r =*.49, *P* < .001).

Bully groups were created using the mean score in the class. “Bullies” scored above the mean on bullying and below the mean on victimization (*n *=* *31; 11%). “Bully‐victims” scored above the mean on both bullying and victimization (*n = *45; 16%). “Noninvolved children” scored below the mean on victimization and scored *zero* on bullying, creating a clear contrast between noninvolved children versus bullies and bully‐victims (*n* = 67; 24%). The remaining children were excluded from the main analyses (*n = *140; 50%).

We conducted sensitivity analyses to investigate whether our results were affected by how we created the bully groups. First, we analyzed the data using stricter criteria to create the bully groups: that is, one standard deviation above the mean (*n* = 17 bullies; *n* = 8 bully‐victims) or the 85th percentile (*n* = 19 bullies; *n* = 7 bully‐victims). Second, we analyzed the data using nomination scores as continuous variables, enabling us to include data of all children. Using regression analyses, we tested whether the main effect of bullying on each psychological process was moderated by victimization, which would indicate that children who scored high on bullying and victimization (i.e., “bully‐victims”) had different scores than children who scored high on bullying but low on victimization (i.e., “bullies”). Third, we analyzed the data excluding classes with nomination rates lower than 60% (instead of 50%). All of these sensitivity analyses yielded the same conclusions as our main analysis (see supplementary material).

#### 2.3.2 Theory of mind errors

Theory of mind errors were assessed using two variants of standard false‐belief tasks (for a detailed description see Hughes et al., 2000). The first task assessed first‐order false‐belief and belief‐emotion reasoning. Children were introduced to a plush rabbit that really liked smarties. Next, the rabbit left. Children were shown a smarties box and learned that this box actually contained grit instead of smarties. Upon the rabbit's return, children were asked what the rabbit would think was inside the box (first order false‐belief question) and what really was inside the box (reality control question). Next, the experimenter gave the box to the rabbit, and children were asked if the rabbit would feel happy or not happy upon getting the box (belief‐desire question); this question was repeated after the rabbit had opened the box (emotion control question).

The second task assessed first‐ and second‐order false‐belief, and was acted out by the experimenter using toy figurines and a toy house. Children saw a girl putting her ball into a red box. After she left the house, another girl changed the location of the ball from the red box to a blue box. Children were then asked where the girl who went outside would think her ball was (first‐order false‐belief question) and where the ball really was (reality control question). Next, the experimenter showed that the girl who went outside had looked through the window and had seen the other girl move her ball. The girl then returned into the house and children were asked in which box she would search according to the girl who moved the ball (second‐order false‐belief question) and where the ball really was (reality control question).

Children's responses on the four theory of mind questions were scored as *correct* (score = 0) if they answered both this question and the corresponding control question correctly, and as *incorrect* (score = 1) if they erred on this question but answered the control question correctly. Responses were coded as missing, if children erred on the control question. Scores were averaged to create a single theory of mind error score. We calculated Cronbach's α for categorical items using categorical principal components analysis (CATPCA; Meulman, Van Der Kooij, & Heiser, 2004). Reliability was sufficient (*α* =.69).

#### 2.3.3 Hostile intent attributions

Hostile intent attributions were assessed using four vignettes describing a hypothetical interaction between the child and a same‐sex protagonist. The vignettes described ambiguous provocations—that is the protagonist caused a bad outcome, but it was unclear whether this bad outcome was intended (Feshbach, 1989). Story themes were provocations familiar to young children: (1) being hurt; (2) a drawing being ruined; (3) being refused to join a game; and (4) a toy being taken. The stories were read aloud by the experimenter and each was accompanied by three 8 × 8 cm black‐and‐white line drawings. Following each vignette, children were asked two questions to assess their intent attributions. First, children were asked why the protagonist had caused the bad outcome. If their response did not reflect a hostile or a benign attribution, they were asked a forced‐choice question (37% of responses; e.g., “did the girl try to ruin your drawing or did she not pay attention?”). Second, children were asked whether the protagonist was trying to be *mean* or *not mean*.

Children's intent attributions were coded by two of the female research assistants into the following categories: (a) *hostile intent*, if children indicated that harm was caused on purpose (15%, e.g., “he was jealous of my drawing”); (b) *benign intent*, if children indicated that harm was caused by accident (58%, e.g., “I was sitting too close to her arm”); or (c) *unclear*, if children made both hostile and benign intent attributions, merely remarked upon the story, or did not answer (27%, e.g., “her arm slipped or she wanted to bump me,” “she is sad”). All responses were coded twice and inter‐coder reliability was good (*M*_κ_ =.84, range =.78–.90). Coding disagreements (8% of responses) were resolved by discussion, using children's scores on the forced‐choice probe question when available. *Hostile intent* and *mean* responses were coded as 1; *benign intent* and *not mean* responses were coded as 0. These scores were averaged over the eight questions to create a single hostile intent attribution score. Reliability for categorical items was sufficient: *α* =.71.

#### 2.3.4 Happy victimizer emotions

Happy victimizer emotions were assessed using four vignettes describing moral transgressions between two same‐sex children of the target child's age (Malti, Gummerum, Keller, & Buchmann, 2009). Two stories described the omission of a prosocial duty (i.e., not sharing a pencil with another child; not helping another child who had fallen) and two stories described doing harm (i.e., stealing another child's chocolate; pushing another child off the swing). The stories were read aloud by the experimenter and each was accompanied by three 8 × 8 cm black‐and‐white drawings. Following each vignette, children were asked whether the transgressor's behavior was “okay or not okay?” In line with previous research, all children knew that it was not okay (99% of responses). Next, to assess children's anticipated emotions, they were asked how they would feel if they had been the transgressor in the vignette story. If their answer did not refer to emotions or described neutral or mixed emotions (23% of responses), they were asked a forced‐choice probe question: “would you feel happy or not happy?”

Children's anticipated emotions were coded by two of the research assistants (other than the assistants who coded children's intent attributions) into the following categories: (a) *positive emotions* (42%; e.g., “happy,” “good,” “fine”); (b) *negative emotions* (40%; e.g., “bad,” “guilty,” “ashamed”); or (c) *other* (18%; e.g., “just okay,” “cheeky”). All responses were coded twice and inter‐coder reliability was good (*M*_κ_ =.95, range =.93–.99). Coding disagreements (2% of responses) were resolved by discussion. For each vignette, children received a score of 1 if they anticipated *positive emotions*, and a score of 0 if they anticipated *negative emotions*. These scores were averaged over the four vignettes to create a single happy victimizer score. Reliability for categorical items was good: *α* =.87.

#### 2.3.5 Reactive and proactive motives

Reactive and proactive motives were assessed using the Instrument for Reactive and Proactive Aggression (IRPA; Polman et al., 2009). Teachers rated the frequency of seven forms of aggressive behavior (i.e., kicking, pushing, hitting, name calling, arguing, gossiping, and doing sneaky things) within the last week, on a 5‐point Likert scale (0 = *never*, 1 = *once*, 2 = *several times*, 3 = *every day*, 4 = *several times a day*). Next, for each form of aggressive behavior that occurred at least once (score > 0), teachers rated three items on reactive motives (e.g., “because this child was angry”) and three items on proactive motives (e.g., “because this child wanted to dominate others”) on a 5‐point Likert scale (0 = *never*, 1 = *rarely*, 2 = *sometimes*, 3 = *most of the times*, 4 = *always*). Thus, both reactive and proactive motive scales had 21 items (i.e., three motive items for each of seven behavior items).

Scores for both on reactive and proactive motives were calculated as the average across the 21 items. Children who never showed any form of aggression received a score of 0 on both motive scales. The internal consistency of the scales was good (reactive motives: *α* = 85; proactive motives: *α* =.79). Scores on the frequency of aggressive behavior were calculated by averaging teachers’ ratings on the seven behavior items (*α* =.79).

### 2.4 Data analysis strategy

We tested our two contrasting hypotheses using Bayesian Inequality and Equality constrained Model Selection (BIEMS), applying the software's default settings (i.e., objective priors; Mulder, Hoijtink, & de Leeuw, 2012). Using BIEMS enabled us to test our hypotheses as coherent models (instead of testing differences between bullies and bully‐victims for each psychological process separately). Thus, this statistical approach enabled us to conduct a single test that directly indicates which hypothesis receives most support from the data.

First, each hypothesis was translated into a model using inequality constraints on the means to specify the expected differences between the bully groups for each variable. For instance, the distinct processes hypothesis predicts that bully‐victims make more theory of mind errors than both bullies and noninvolved children, which was specified with the inequality constraints [*M* bully‐victim > *M* bully] and [*M* bully‐victim > *M* noninvolved]. In contrast, the shared processes hypothesis predicts that both bullies and bully‐victims make more theory of mind errors than noninvolved children, which was specified as [*M* bully > *M* noninvolved] and [*M* bully‐victim > *M* noninvolved].

Next, we evaluated these models. Bayesian model selection does not rely on significance testing or *P‐*values but instead computes Bayes factors that quantify to what extent the data support one model compared to another. A Bayes factor of 1 indicates equal support for both models; a Bayes factor of >1 indicates support in favor of one model over another (e.g., a Bayes factor of *BF_modelA > B_ = *4 indicates that this model A received four times more support from the data than model B). Before testing our hypotheses, we first tested whether each model had a sufficient fit to the data by computing a Bayes factor of the model against the unconstrained (null) model. Next, we tested our two hypotheses against each other by computing the Bayes factor between the two models.

## 3 RESULTS

### 3.1 Preliminary analyses

#### 3.1.1 Correlations

Zero‐order correlations between the study variables are shown in Table 2. First, as expected, we found negative correlations of age with theory of mind errors, hostile intent attributions, and happy victimizer emotions. Second, most psychological processes were positively correlated to the bully or victim nomination scores—with the exception of happy victimizer emotions, which was related to neither. Third, the correlation between reactive and proactive motives was lower than typically reported in questionnaire studies (i.e., *r =*.43, compared to *r* =.70 as found in a meta‐analysis; Polman et al., 2007). This relatively low correlation enabled us to study distinct motives in bully and bully‐victim groups, beyond these children's shared tendency to aggress.

**Table 2:** Zero‐order correlations between the study variables for the complete sample (*N = *283)

	Sex	Age	B	V	RE	PRO	TOM	HIA	HV
*n* =	283	283	237	237	283	283	280	282	282
Bully nominations (B)	−.37***	.11	–						
Victim nominations (V)	.04	−.21**	.18**	–					
Reactive motives (RE)	−.22***	.05	.45***	.25***	–				
Proactive motives (PRO)	−.08	−.06	.40***	.15*	.43***	–			
Theory of mind errors (TOM)	−.00	−.56***	.02	.13*	−.04	.01	–		
Hostile intent attributions (HIA)	−.11	−.24***	.13*	−.09	.02	.03	.24***	–	
Happy victimizer emotions (HV)	.05	−.15*	−.06	−.02	−.05	−.05	.01	.05	–

Missing scores (i.e., n ≠ 283) indicate that children failed to complete a task (TOM, HIA, HV) or were in a class with <50% participation rate (B, V).

*P < .05, **P < .01, ***P < .001.

#### 3.1.2 Bully groups

Table 3 shows descriptive statistics for the three bully groups. As can be seen, boys were overrepresented in the bully and bully‐victim groups, whereas girls were overrepresented in the noninvolved group, *χ* ^2^ = 27.08, *P *< .001. There were no significant age differences between the bully groups, *F*(2, 140) = 1.68, *P =*.190. A MANOVA indicated that the three bully groups differed in teacher‐rated frequency of aggressive behavior, bully nominations, and victim nominations, *F*(6, 278) = 38.52, *P *< .001. Post‐hoc tests showed that bully‐victims received more victim nominations than bullies, but received similar numbers of bully nominations and aggression ratings (Table 3), indicating that bullies and bully‐victims were comparable in their levels of bullying and aggressive behavior.

**Table 3:** Means (and standard deviations) for age, aggression ratings, bully and victim nominations, and the number (and %) of boys and girls nominated as bully, bully‐victim, or noninvolved

	Bully (*n* = 31)	Bully‐victim (*n* = 45)	Noninvolved (*n* = 67)
Boys	25 (26.3)	40 (42.1)	30 (31.6)
Girls	6 (12.5)	5 (10.4)	37 (77.1)
Age (in years)	6.76 (1.31)	7.21(1.23)	6.80(1.31)
Aggression ratings	0.59^a^ (0.64)	0.49^a^ (0.61)	0.11^b^ (0.22)
Bully nominations	0.36^a^ (0.21)	0.32^a^ (0.18)	0.00^b^ (0.00)
Victim nominations	0.17^b^ (0.12)	0.37^a^ (0.14)	0.12^b^ (0.08)

Groups with different superscripts differ significantly at *α *< .01.

#### 3.1.3 Sex and age differences

To explore whether the results for the primary analyses differed for boys and girls or by age, we analyzed interaction effects of sex and age with bully group for each psychological process. No moderation effects were found (all *p*s > .05).

### 3.2 Primary analyses

Table 4 shows means and standard deviations on the psychological process variables for children nominated as bully, bully‐victim, and noninvolved. The mean differences indicate that bullies and bully‐victims both had higher scores on reactive and proactive motives than noninvolved children; that bullies had higher scores on hostile intent than bully‐victims and noninvolved children; and that there were no differences between the bully groups on theory of mind errors and happy victimizer emotions.

**Table 4:** Means (and standard deviations) of psychological process variables for children nominated as bully, bully‐victim, or noninvolved

	Bully (*n *= 31)	Bully‐victim (*n *= 45)	Noninvolved (*n *= 67)
Reactive motives	1.50^a^ (0.94)	1.42^a^ (1.09)	0.42^b^ (0.75)
Proactive motives	1.02^a^ (0.93)	0.89^a^ (1.06)	0.22^b^ (0.49)
Theory of mind errors	0.14^a^ (0.20)	0.10^a^ (0.17)	0.13^a^ (0.24)
Hostile intent attributions	0.45^a^ (0.30)	0.29^b^ (0.26)	0.29^b^ (0.23)
Happy victimizer emotions	0.43^a^ (0.41)	0.43^a^ (0.41)	0.57^a^ (0.42)

Groups with different superscripts differ significantly at *α *< .01.

To test whether these results—as a pattern—are more in line with the distinct processes hypothesis or the shared processes hypothesis, we used BIEMS to run these two models on the data. First, we assessed the fit of each model by comparing it to the unconstrained (null) model. This analysis yielded *BF*_distinct_ **< 0.01 and *BF*_shared_ *= *0.45. Both Bayes factors were below 1, so this result indicates that neither hypothesis received more support from the data than the unconstrained (null) model.

Next, we reran the analysis excluding happy victimizer emotions, as this variable was unrelated to both bully and victim nominations. This second analysis yielded *BF*_distinct_ = 0.03 and *BF*_shared_ *= *7.46, indicating that the shared processes model received more support from the data than the unconstrained (null) model, whereas the distinct processes model did not. Last, we computed the evidence for the shared processes model over the distinct processes model by dividing these Bayes factors, yielding *BF*_shared>disctinct_ = 248.67: the shared processes hypothesis received over two hundred times more support from the data than the distinct processes hypothesis.

## 4 DISCUSSION

The present study tested two contrasting hypotheses, examining whether young “bullies” and “bully‐victims” have distinct or shared psychological processes underlying their bullying behavior (Table 1). The “distinct processes hypothesis” predicts that bullies have proactive motives for aggression and anticipate happiness after victimizing others, whereas bully‐victims have reactive motives for aggression, poor theory of mind skills, and attribute hostile intent to others. In contrast, the “shared processes hypothesis” predicts that bullies and bully‐victims deviate on all psychological processes alike. We analyzed our results using Bayesian model selection, enabling us to conduct a single test to compare our two hypotheses. The data provided 249 times more support for the shared processes hypothesis than for the distinct processes hypothesis.

Taken together, these findings suggest that, at an early age bullies and bully‐victims have shared, rather than distinct, psychological processes underlying their bullying behavior. The support for the shared processes hypothesis implies that psychological processes may not so much predict from which social position children engage in bullying (i.e., bully or bully‐victim), but rather whether children engage in bullying at all. Notably, both bullies and bully‐victims resort to aggressive strategies when interacting with peers (rather than resorting to avoidant or prosocial strategies, for instance). But if bullies and bully‐victims have shared psychological processes, what then explains their different social positions (i.e., bullies are typically well‐integrated, whereas bully‐victims are typically marginalized; Farmer et al., 2010; Perren & Alsaker, 2006)? Possibly, these different social positions are explained by young children's success with behaving aggressively: Bullies may be the children who have gained dominance by behaving aggressively, whereas bully‐victims may be the children who have evoked victimization by behaving aggressively (Perren & Alsaker, 2006). Thus, one potential explanation for our findings is that bullies and bully‐victims do not differ in their motives for aggression at this early age, but rather in the successfulness of their aggression.

It is important to note that this interpretation is limited to early childhood; it is possible that in later childhood bullies and bully‐victims do have distinct psychological processes underlying their bullying behavior. Throughout this paper, we have conceptualized psychological processes as antecedents of bullying behavior; however, many of the processes studied may as well be the consequences of bullying or victimization. For instance, children who are repeatedly victimized may over time develop a tendency to attribute hostile intent. Thus, it is possible that stable psychological characteristics predicting children's position as bully or bully‐victim may emerge later in childhood.

This study found that bullies and bully‐victims in early childhood are similar in their reactive and proactive motives for aggression, replicating previous research in adolescence (Bettencourt & Farrell, 2013; Ragatz et al., 2011; Salmivalli & Nieminen, 2002). This finding is important, because the notion of distinct motives for aggression is at the heart of current theorizing about other distinct psychological processes in bullies and bully‐victims. Instead, if the aggressive behavior of bullies and bully‐victims is similarly motivated, there is less reason to presume other differences—for instance, that bullies expect to feel happy after victimizing whereas bully‐victims expect their peers to have hostile intentions.

Indeed, we did not find the predicted differences between bullies and bully‐victims on theory of mind skills, hostile intent attributions, and happy victimizer emotions. However, we also did not find differences between noninvolved children versus bullies and bully‐victims, as predicted by both hypotheses. This was unexpected, because we selected our psychological processes for their known relevance to predict aggressive behavior in young children (de Castro, Veerman, Koops, Bosch, & Monshouwer, 2002; Malti & Krettenauer, 2013; Sutton et al., 1999). Moreover, these processes all correlated with age in expected directions (i.e., resonating with children's social and moral development in early childhood; Flavell, 1999; Malti & Ongley, 2014).

Concerning theory of mind skills, ours is not the first study to find no differences between bullies and noninvolved children in early childhood (e.g., Gini, 2006; Monks, Smith, & Swettenham, 2005; Sutton et al., 1999). Yet, one longitudinal study did find evidence for poorer theory of mind skills at age 5 in children subsequently identified as bully or bully‐victim age 12 (Shakoor et al., 2012). These findings suggest that the detrimental effects of poor theory of mind skills may build up during years of peer interactions and may only manifest themselves in bullying behavior in later childhood.

Concerning hostile intent attributions, our results were puzzling. We found that bullies make more hostile intent attributions than bully‐victims, whereas the distinct processes hypothesis predicts the opposite pattern: that bully‐victims make more hostile intent attributions than bullies. As bullies and bully‐victims were equally aggressive according to teachers, this finding cannot be explained by bullies showing more severe behavior. Rather, these results may be a chance finding: There were three children who had the highest possible score on hostile intent attributions and these children were all categorized as bully. Indeed, if we excluded data of these children from the multivariate analysis, the difference between bullies and bully‐victims became nonsignificant. It is possible that these children's extreme scores were outliers or stemmed from home experiences such as harsh parenting (Weiss, Dodge, Bates, & Pettit, 1992). However, these interpretations are quite speculative and further research seems warranted.

The variable happy victimizer emotions was unrelated to both bullying and victimization and was removed from the analyses in order to obtain adequate model fit. One explanation for this finding is that happy victimizer emotions are not as relevant for predicting bullying (Gini, 2006; Menesini & Camodeca, 2008) as they are for predicting aggressive behavior (Malti & Krettenauer, 2013). Alternatively, happy victimizer emotions may be relevant for predicting bullying behavior but only when assessed in a more fine‐grained manner: Some bullies may attribute negative emotions merely because they expect to be sanctioned, but would attribute happiness if they expected their victimization to remain undetected (as is often the case for bullying behavior). Research that additionally assesses children's reasoning behind their anticipated emotions may be more sensitive to detect subtle differences in children's motivations for bullying (Nunner‐Winkler, 2007). In addition, research that directly compares different ages may provide further information about developmental differences and similarities in relations between happy victimizer emotions and bullying behavior.

Collectively, the results show the advantage of using an integrative approach to study several psychological processes in bullies and bully‐victims (Malti, 2016). When considering each process separately, the results yielded mixed evidence; however, when using Bayesian model selection to compare our two hypotheses as coherent models, the data clearly favored the shared processes hypothesis. This is one important illustration of why researchers in social science have recommended the use of Bayesian statistics (Van de Schoot et al., 2014).

Our findings are based on comparisons between bully groups and so it is important to carefully consider how these groups were created. First, we assigned children to a bully group if they received more nominations than the mean of their classroom. This approach yielded sufficient group sizes, but lower severity of bullying and victimization problems within the groups. However, our results do not seem to be affected by this approach: we found similar results using different criteria to create groups. Second, we used victim nominations to discriminate bullies from bully‐victims. A previous study found that kindergarteners gave more victim nominations to their friends, casting some doubt regarding the validity of such nominations (Monks, Smith, & Swettenham, 2003). In our study, however, it seems unlikely that victim nominations reflected friendship because they were correlated with several variables indicative of low social competence (i.e., poor theory of mind, and reactive and proactive motives for aggression). Third, we based our groups solely on peer‐nominations. We refrained from using combined peer‐ and teacher‐report to avoid shared informant bias: Teachers’ perceptions of bullying and victimization may generalize to their report of reactive and proactive motives, and vice versa. To enhance the reliability of our peer‐nomination assessment, we used an interview developed for use with kindergarteners; including pictures to explain the definition of bullying, and photographs of classmates to help children with recognition (Alsaker et al., 2008). We have indications this worked out sufficiently: there were meaningful associations between the peer‐nomination scores and psychological process variables, and the bully groups corroborated with teacher‐ratings of aggression.

In sum, we find that young bullies and bully‐victims have shared psychological processes underlying their bullying behavior. These findings raise new questions concerning what exactly differentiates bullies from bully‐victims: Is their behavior differently motivated, or do they differ in the successfulness of their aggression? Should psychological processes be regarded as the antecedents of children's position as bully or bully‐victim, as the consequences, or both (but in different developmental stages)? Thus, the present study has set a starting point for future research by clarifying that, in early childhood, bullies and bully‐victims have shared rather than distinct psychological processes underlying their behavior.

## Supporting information

Additional Supporting Information may be found online in the supporting information tab for this article.

Supporting Information S1.

## ENDNOTE

1 Raw data, analysis code, and relevant study materials are available at: https://osf.io/tzjwk
