# Supplementary material for: Psychological processes in young bullies versus bully‐victims
Source: Aggress Behav. 2017 Feb 8;43(5):430–9. doi: 10.1002/ab.21701 (PMC5573968; doi:10.1002/ab.21701)
Supplement: Supplementary file 1 — Supporting Information S1. [file AB-43-430-s001.docx]

**Supplementary Material**

**Sensitivity analyses for bully group calculations**

We conducted three sensitivity analyses to investigate whether our results were affected by how we created the bully groups: (1) one standard deviation above the mean; (2) the 85^th^ percentile; (3) excluding classes with nomination rates lower than 60% (instead of 50%). Table S1 shows the mean scores per bully group on our psychological process variables for each grouping approach. We reran the Bayesian model selection, our primary analysis, on the group means obtained with the three different grouping approaches. The results were highly similar to the mean-split approach reported in the paper (i.e., *BF*_distinct_ = 0.03 and *BF*_shared_*=*7.46): For the standard deviation approach we found *BF*_distinct_ = 0.10 and *BF*_shared_*=*6.66; and for the 85^th^ percentile approach we found *BF*_distinct_ = 0.62 and *BF*_shared_ *=*7.81; and for the >60% approach we found *BF*_distinct_ = 0.74 and *BF*_shared_*= 2*6.20.

The results for each psychological process separately were also similar to the results reported in the paper, with the one exception that the standard deviation approach yielded higher (instead of similar) mean scores for bully-victims versus bullies on reactive motives and proactive motives. However, this may be a chance finding because it was obtained only for one out of three grouping approaches. Moreover, this finding did not affect our main conclusion, because it was as much in line with the distinct processes hypothesis (i.e., more reactive motives in bully-victims than bullies) as it was in contrast with this hypothesis (i.e., more proactive motives in bully-victims than bullies). In sum, our sensitivity analyses yielded results highly similar to the results reported in the manuscript, suggesting that our conclusions are not dependent on the specific way in which the groups were created.

**Analyses using continuous scores instead of bully groups**

We conducted a set of analyses using continuous scores for bullying and victimization instead of creating bully groups. We ran a regression analysis for each dependent variable including age and sex in the first block, bullying and victimization scores in the second block, and the interaction between bullying and victimization in the third block. The results of these analyses lead to the same conclusions as the categorical approach reported in the paper (Table S2): For theory of mind and happy victimizer emotions, we found no main effect of bullying nor an interaction with victimization—similar to the finding that bullies, bully-victims, and noninvolved children did not score differently on these variables; For hostile intent attributions, we found a main effect of bullying and an interaction with victimization, indicating that bullying only predicted hostile intent attributions for children low on victimization—similar to the finding that bullies made more hostile intent attributions than bully-victims; For reactive and proactive motives, we found a main effect of bullying but no interaction with victimization—similar to the finding that bullies and bully-victims scored higher than noninvolved children on these variables, but did not differ from each other. These analyses suggest that the results reported in the paper do not depend on our approach to data analysis.

Table S1

Means (*M*) and Standard Deviations (*SD*) of Psychological Process Variables for Children Nominated as Noninvolved, Bully-Victim, or Bully Based on Different Grouping Criteria

|  |  | As in paper | | | *M* ± 1 *SD* | | | 85^th^ percentile | | | >60% participation | | |
| --- | --- | --- | --- | --- | --- | --- | --- | --- | --- | --- | --- | --- | --- |
|  |  | *M* | *SD* | *n* | *M* | *SD* | *n* | *M* | *SD* | *n* | *M* | *SD* | *n* |
| TOM | Noninvolved | 0.13^a^ | 0.24 | 67 | 0.12^a^ | 0.23 | 104 | 0.12^a^ | 0.21 | 193 | 0.08^a^ | 0.15 | 54 |
|  | Bully-victim | 0.10^a^ | 0.17 | 45 | 0.00^a^ | 0.00 | 8 | 0.00^a^ | 0.00 | 7 | 0.08^a^ | 0.15 | 36 |
|  | Bully | 0.14^a^ | 0.20 | 31 | 0.20^a^ | 0.23 | 17 | 0.18^a^ | 0.23 | 19 | 0.12^a^ | 0.20 | 23 |
| HIA | Noninvolved | 0.29^a^ | 0.23 | 67 | 0.31^a^ | 0.23 | 104 | 0.29^a^ | 0.25 | 193 | 0.26^a^ | 0.22 | 54 |
|  | Bully-victim | 0.29^a^ | 0.26 | 45 | 0.17^a^ | 0.18 | 8 | 0.23^a^ | 0.15 | 7 | 0.26^a^ | 0.23 | 36 |
|  | Bully | 0.45^b^ | 0.23 | 31 | 0.48^b^ | 0.32 | 17 | 0.44^b^ | 0.34 | 19 | 0.43^b^ | 0.30 | 23 |
| HV | Noninvolved | 0.57^a^ | 0.42 | 67 | 0.58^a^ | 0.42 | 104 | 0.50^a^ | 0.43 | 193 | 0.57^a^ | 0.43 | 54 |
|  | Bully-victim | 0.43^a^ | 0.41 | 45 | 0.50^a^ | 0.42 | 8 | 0.54^a^ | 0.44 | 7 | 0.43^a^ | 0.42 | 36 |
|  | Bully | 0.43^a^ | 0.41 | 31 | 0.38^a^ | 0.45 | 17 | 0.50^a^ | 0.41 | 19 | 0.41^a^ | 0.42 | 23 |
| RE | Noninvolved | 0.42^a^ | 0.75 | 67 | 0.53^a^ | 0.82 | 104 | 0.70^a^ | 0.92 | 193 | 0.39^a^ | 0.75 | 54 |
|  | Bully-victim | 1.42^b^ | 1.09 | 45 | 2.51^b^ | 0.70 | 8 | 2.40^b^ | 1.08 | 7 | 1.55^b^ | 1.12 | 36 |
|  | Bully | 1.50^b^ | 0.94 | 31 | 1.35^c^ | 0.97 | 17 | 1.67^b^ | 0.89 | 19 | 1.35^b^ | 0.95 | 23 |
| PRO | Noninvolved | 0.22^a^ | 0.49 | 67 | 0.35^a^ | 0.64 | 104 | 0.44^a^ | 0.76 | 193 | 0.22^a^ | 0.51 | 54 |
|  | Bully-victim | 0.89^b^ | 1.06 | 45 | 1.56^b^ | 1.18 | 8 | 1.03^ab^ | 0.83 | 7 | 1.05^b^ | 1.12 | 36 |
|  | Bully | 1.02^b^ | 0.93 | 31 | 0.92^c^ | 0.91 | 17 | 1.30^b^ | 0.98 | 19 | 1.17^b^ | 0.97 | 23 |

*Note.* Groups with different superscripts differ significantly at α < .01. TOM = theory of mind errors, HIA = hostile intent attributions, HV = happy victimizer emotions, RE = reactive motives, PRO = proactive motives.

Table S2

Regressions Predicting Children’s Theory of Mind Errors (TOM), Hostile Intent Attributions (HIA), Happy Victimizer Emotions (HV), Reactive Motives (RE), and Proactive Motives (PRO): Bully and Victim Nominations on Step 2 and the Interaction Effect on Step 3

|  |  | TOM | | HIA | | HV | | RE | | PRO | |
| --- | --- | --- | --- | --- | --- | --- | --- | --- | --- | --- | --- |
| Step | | *R*^2^ | β | *R*^2^ | β | *R*^2^ | β | *R*^2^ | β | *R*^2^ | β |
| 1. | Age | .53^***^ | -.53^***^ | .26^***^ | -.24^***^ | .16^†^ | -.12^†^ | .21^**^ | -.03 | .10 | -.09 |
|  | Sex |  | .02 |  | -.10 |  | .10 |  | -.21^**^ | .43^***^ | -.05 |
| 2. | Bully (B) | .54 | .09 | .34^**^ | .19^**^ | .17 | -.00 | .48^***^ | .39^***^ |  | .44^***^ |
|  | Victim (V) |  | -.00 |  | -.19^***^ |  | -.05 |  | .17^**^ |  | .04 |
| 3. | B×V | .54 | -.05 | .36^*^ | -.13^*^ | .17 | .02 | .48 | -.03 | .43 | -.02 |

*Note.* Standardized betas and *R*^2^ are taken from each step; significance values for *R*^2^ represent change. ^†^*p* < .10; ^*^*p* < .05; ^**^*p* < .01; ^***^*p* < .001.
